# PSMA-ligand uptake can serve as a novel biomarker in primary prostate cancer to predict outcome after radical prostatectomy

**DOI:** 10.1186/s13550-021-00818-2

**Published:** 2021-08-21

**Authors:** Hui Wang, Thomas Amiel, Christoph Würnschimmel, Thomas Langbein, Katja Steiger, Isabel Rauscher, Thomas Horn, Tobias Maurer, Wolfgang Weber, Hans-Juergen Wester, Karina Knorr, Matthias Eiber

**Affiliations:** 1 Department of Nuclear Medicine, Klinikum rechts der Isar, Technical University Munich, Ismaninger Str. 22, 81675 Munich, Germany; 2 Department of Urology, Klinikum rechts der Isar, Technical University Munich, Ismaninger Str. 22, 81675 Munich, Germany; 3 Martini-Klinik Prostate Cancer Center, University Hospital Hamburg-Eppendorf, Martinistr. 52, 20246 Hamburg, Germany; 4 Institute of Pathology, School of Medicine, Technical University Munich, Trogerstr. 18, 81675 Munich, Germany; 5 Department of Urology, University Hospital Hamburg-Eppendorf, Martinistr. 52, 20246 Hamburg, Germany; 6 Pharmaceutical Radiochemistry, Technical University of Munich, Walther-Meißner-Str. 3, 85748 Garching, Germany

**Keywords:** Biochemical recurrence, ^68^Ga-PSMA-11 PET, miTNM classification, Prostate cancer

## Abstract

**Background:**

The prostate-specific membrane antigen (PSMA) is a relevant target in prostate cancer, and immunohistochemistry studies showed associations with outcome. PSMA-ligand positron emission tomography (PET) is increasingly used for primary prostate cancer staging, and the molecular imaging TNM classification (miTNM) standardizes its reporting. We aimed to investigate the potential of PET-imaging to serve as a noninvasive imaging biomarker to predict disease outcome in primary prostate cancer after radical prostatectomy (RP).

**Methods:**

In this retrospective analysis, 186 primary prostate cancer patients treated with RP who had undergone a ^68^Ga-PSMA-11 PET up to three months prior to the surgery were included. Maximum standardized uptake value (SUV_max_), SUV_mean_, tumor volume (TV) and total lesion (TL) were collected from PET-imaging. Moreover, clinicopathological information, including age, serum prostate-specific antigen (PSA) level, and pathological characteristics, was assessed for disease outcome prediction. A stage group system for PET-imaging findings based on the miTNM framework was developed.

**Results:**

At a median follow-up after RP of 38 months (interquartile range (IQR) 22–53), biochemical recurrence (BCR) was observed in 58 patients during the follow-up period. A significant association between a positive surgical margin and miN status (miN1 vs. miN0, odds ratio (OR): 5.428, *p* = 0.004) was detected. miT status (miT ≥ 3a vs. miT < 3, OR: 2.696, *p* = 0.003) was identified as an independent predictor for Gleason score (GS) ≥ 8. Multivariate Cox regression analysis indicated that PSA level (hazard ratio (HR): 1.024, *p* = 0.014), advanced GS (GS ≥ 8 vs. GS < 8, HR: 3.253, *p* < 0.001) and miT status (miT ≥ 3a vs. miT < 3, HR: 1.941, *p* = 0.035) were independent predictors for BCR. For stage I disease as determined by PET-imaging, a shorter BCR-free survival was observed in the patients with higher SUV_max_ (IA vs. IB stage, log-rank, *p* = 0.022).

**Conclusion:**

Preoperative miTNM classification from ^68^Ga-PSMA-11 PET correlates with postoperative GS, surgical margin status and time to BCR. The association between miTNM staging and outcome proposes ^68^Ga-PSMA-11 PET as a novel non-invasive imaging biomarker and potentially serves for ancillary pre-treatment stratification.

**Supplementary Information:**

The online version contains supplementary material available at 10.1186/s13550-021-00818-2.

## Background

Approximately 30% to 40% of prostate cancer patients will fail primary treatment requiring further disease management [1, 2]. Traditional risk factors, including preoperative serum prostate-specific antigen (PSA) level [3–6], pathological stage [7] and Gleason score (GS) [5–9], are widely used for biochemical recurrence (BCR) prediction. However, there is growing interest in identifying novel biomarkers to improve BCR prediction accuracy of prostate cancer patients after radical prostatectomy (RP).

In the last few years positron emission tomography (PET) probes targeting prostate-specific membrane antigen (PSMA) has significantly improved detection and localization of disease in primary and recurrent prostate cancer [1, 10–12]. PSMA is a type II integral membrane glycoprotein with folate hydrolase activity, internalization after activation and is encoded by the FOLH1 gene [13, 14]. PSMA expression increases progressively in higher-grade prostate tumor cells and metastatic lesions [15, 16].

Increased PSMA expression in immunohistochemistry was more often observed in pathological stage III or IV tumors (51%) compared to stage I and II tumors (32%, *p* = 0.029) [17]. High-level PSMA expression in immunohistochemistry was associated with a higher risk of BCR and overall survival in several studies [16, 17]. Finally, expression of membranous PSMA is also associated with higher rates of defective deoxyribonucleic acid (DNA) damage repair gene [18].

In the last two decades, for different tumor entities results from imaging were introduced as non-invasive quantitative biomarkers. For fluorodeoxyglucose (FDG)-PET-imaging, Wieder et al. have demonstrated that mean standardized uptake value (SUV_mean_) can be used to preoperatively predict histopathological response in esophageal squamous cell carcinoma (ESCC) patients. A decrease in SUV_mean_ of 44% ± 15% from responders and 21% ± 14% from non-responders (*p* = 0.0055) was observed after radiochemotherapy [19]. Besides, Giovacchini et al. have observed that the positive results of ^11^C-choline PET/computed tomography (CT) predict prostate cancer-specific survival in patients after RP [20]. For metastatic castration-resistant prostate cancer (mCRPC), the bone scan index (BSI) [21] and quantitative parameter from PET have been reported to serve as potential predictive biomarkers for bone tumor burden [22, 23].

To standardize reporting of PSMA-targeted PET-imaging, a unified molecular-imaging TNM classification (miTNM, version 1.0) has been recently introduced [24]. It is envisioned that its system classifying tumor extent similar to the pathological TNM-system might serve as qualitative imaging biomarker potentially stratifying disease outcome.

The aim of our retrospective analysis was to investigate the potential of quantitative and qualitative parameters from ^68^Ga-PSMA-11 PET to serve as non-invasive imaging biomarkers to predict BCR in primary prostate cancer after RP allowing for ancillary preoperative risk stratification.

## Methods

### Patient selection

We screened the institutions´ database for all patients who underwent ^68^Ga-PSMA-11 PET imaging maximum 3 months prior the RP between January 2013 and August 2017. All patients who received neoadjuvant therapy prior to RP had PSA-persistence after RP or in whom follow-up data were missing were excluded. Finally, 186 patients with D’Amico intermediate- to high-risk primary prostate cancer were included in this retrospective study (Additional file 1: Fig. S1). Table 1 summarizes the clinical and histopathological characteristics. BCR was defined as a serum PSA level rising above 0.2 ng/ml. The primary endpoint was time to BCR. The time to BCR was calculated from the date of surgery. The retrospective analysis has been approved by the Ethics Committee of the Technical University Munich (750/20 S-KH).

**Table 1:** Patient characteristics

Characteristic	Patients
Age (years), median (IQR), *n* = 186	68 (61–72)
iPSA (ng/ml), median (IQR), *n* = 184^a^	9.7 (6.5–15.1)
Administered ^68^Ga-PSMA-11 activity (MBq), median (IQR), *n* = 185^b^	139 (112–156)
Time PET to RP (day), median (IQR), *n* = 186	26 (13–46)
Gleason score in surgical specimen, no. (%), *n* = 186	
6	11 (5.9%)
7a	63 (33.9%)
7b	59 (31.7%)
8	28 (15.1%)
9	25 (13.4%)
Pathological stage, no. (%), *n* = 186	
pT status	
2a	11 (5.9%)
2b	10 (5.4%)
2c	71 (38.2%)
3a	49 (26.3%)
3b	44 (23.7%)
4	1 (0.5%)
pN status	
0	154 (82.8%)
1	32 (17.2%)
Surgical margin, no. (%), *n* = 180^c^	
Negative	152 (84.4%)
Positive	28 (15.6%)

*iPSA* initial PSA, *IQR* interquartile range, *PET* positron emission tomography, *PSA* prostate-specific antigen, *RP* radical prostatectomy

^a^iPSA of two patients were unavailable

^b^The injected dose of ^68^Ga-PSMA-11 from one patient was unavailable

^c^The status of surgical margin from six patients were unavailable

### Imaging protocol

The synthesis of ^68^Ga-PSMA-11 [25] was described previously [26]. Patients were intravenously injected with a median of 139 MBq of ^68^Ga-PSMA-11 (interquartile range (IQR) 112–156). PET acquisition was started at a median of 54 min (IQR 49–65) after the tracer injection. Nighty-three patients underwent ^68^Ga-PSMA-11 PET/CT using a Biograph mCT flow scanner (Siemens Medical Solutions, Erlangen, Germany), and 93 patients underwent ^68^Ga-PSMA-11 PET/magnetic resonance imaging (MRI) using an integrated whole-body PET/MRI system (Siemens Biograph mMR, Erlangen, Germany). Details on PET/CT and PET/MRI acquisition were described previously [27, 28].

### Imaging analysis

^68^Ga-PSMA-11 PET/CT and ^68^Ga-PSMA-11 PET/MRI images were evaluated by one nuclear medicine physician blinded to the postoperative histopathological results. All lesions were reannotated by two experienced board-certified nuclear medicine physicians. Any focal or diffuse tracer uptake in the prostate or extra-prostatic lesions above the surrounding background and not associated with physiological uptake was considered suspicious for malignancy. One circular region in transaxial slices was drawn over the prostate and over every extra-prostatic lesion automatically adapted to a three-dimensional volume of interest (VOI) using Syngo.Via (Siemens Healthineers, Erlangen, Germany). A 40% isocontour of the SUVmax was used to determine the SUVmean. Typical pitfalls in PSMA-ligand PET including low to moderate PSMA uptake correlated with osteoblastic changes (i.e., fractures or degenerative changes), celiac, and ganglia were taken into consideration [29]. SUV_max_, SUV_mean_, tumor volume (TV), and total lesion (TL) of every VOI were calculated. Prostatic and extra-prostatic lesions were classified according to miTNM classification [24]. In PSMA-PET negative primary tumors, the CT or MRI part of the hybrid PET-exam was used for miTNM stage group determination.

Similar to the structure of the American Joint Committee on Cancer (AJCC) Prostate Cancer Prognostic Stage Groups [30], we established a stage group system using the different grades from the miTNM staging system (Table 2). To allow the discrimination into different risk groups based on the characteristics of the primary tumor, we used a SUV_max_ cut-off of 5.4 to subgroup stage I disease into stage IA and IB. The cut-off was derived from a recent study proposing it the optimal cut-off to distinguish between GS ≤ 7a and GS ≥ 7b [31].

**Table 2:** Proposed miTNM stage groups for ^68^Ga-PSMA-11 PET

Stage Group	miT	miN	miM	SUV_max_
IA	2	0	0	< 5.4
IB	2	0	0	≥ 5.4
IIA	3	0	0	Any
IIB	4	0	0	Any
III	Any	1 and 2	0	Any
IV	Any	Any	1	Any

*PET* positron emission tomography, *PSMA* prostate-specific membrane antigen, *SUV* standardized uptake value

### Statistical analysis

Descriptive statistics were used to display continuous variables as the median and IQR with 25th and 75th percentiles (Q1–Q3), mean ± standard deviation (SD), as well as percentages. The association between pathological results and ^68^Ga-PSMA-11 PET findings was investigated with uni- and multivariate Logic regression analyses, and the corresponding odds ratios (OR) and 95% confidence intervals (CI) were calculated. Postoperative BCR-free survival was estimated using the Kaplan–Meier method and compared between groups using the Log-rank test. Moreover, uni- and multivariable Cox regression analysis were performed to determine the ability of clinicopathological factors and ^68^Ga-PSMA-11 PET findings to predict BRC after RP, and the corresponding hazard ratios (HR) and 95% CI were calculated. The multivariable model only included parameters with a significant association on univariable analysis. A *p* value of 0.05 was used as the cut-off for statistical significance.

Given its low sample size (*n* = 7) the miM1 subgroup was excluded for univariable and multivariable analysis.

Statistical evaluation was performed with IBM SPSS Statistics Version 20 (Armonk, NY, USA), and the figures were generated using GraphPad Prism Version 8 (San Diego, California, USA).

## Results

### Histopathological patient characteristics

On post-operative histopathology, a total of 133 (71.5%) patients had a GS < 8, and 53 (28.5%) of the patients had a GS 8 or 9. Lymph node metastases were detected in 32 (17.2%) patients. pT3a, pT3b and pT4 disease was present in 49 (26.3%), 44 (23.7%) and 1 (0.5%) patient, respectively. Twenty-eight (15.6%) patients had positive surgical margins (R1) (Table 1). At a median follow-up of 38 months (IQR 22–53), BCR was observed in 58 (31.2%) patients during the follow-up period.

### ^68^Ga-PSMA-11 PET findings

#### miTNM staging and miTNM stage groups

In 67.2% (*n* = 125) of patients, the primary tumor was classified as miT2, 90.3% (*n* = 168) were classified as miN0, 3.8% (*n* = 7) were classified as miN1, 5.9% (*n* = 11) were classified as miN2, and 96.2% (*n* = 179) were classified as miM0. ^68^Ga-PSMA-11 PET findings (SUV_max_, SUV_mean_, TV, TL) of prostatic lesions were analyzed with 183 patients because three patients were reported negative PSMA prostate cancer. Table 3 lists information from ^68^Ga-PSMA-11 PET. In three cases with PSMA-PET negative primary tumor cross sectional imaging was used to determine the miTNM stage.

**Table 3:** ^68^Ga-PSMA-11 PET findings

Characteristic	Patients
miTNM classification, no. (%), *n* = 186	
miT status	
2u	73 (39.2%)
2m	52 (28%)
3a	27 (14.5%)
3b	24 (12.9%)
4	10 (5.4%)
miN status	
0	168 (90.3%)
1	7 (3.8%)
2	11 (5.9%)
miM status	
0	179 (96.2%)
1a	3 (1.6%)
1b	4 (2.2%)
PSMA-PET findings of prostatic lesions, median (IQR), *n* = 183^a^	
SUV_max_	10.6 (6.4–18.9)
SUV_mean_	6.2 (3.2–11.0)
TV	3.9 (1.7–10.5)
TL	24.7 (15.9–44.4)

*IQR* interquartile range, *PET* positron emission tomography, *PSMA* prostate-specific membrane antigen, *SUV* standardized uptake value, *TL* total lesion, *TV* tumor volume

^a^Three patients had PSMA negative prostate cancer. miTNM of these patients were classified based on MRI images

Based on the proposed stage group system combining the miTNM staging and SUV_max_ of 20 (10.8%), 96 (51.6%), 40 (21.5%), 7 (3.8%), 16 (8.6%) and 7 (3.8%) into the stage groups IA, IB, IIA, IIB, III and IV was performed, respectively.

#### Correlation of 68Ga-PSMA-11 PET parameters with histopathology

The sensitivity and specificity of ^68^Ga-PSMA-11 PET detecting pelvic lymph nodes metastasis were 40.6% and 96.8% (13/32 and 149/154, respectively). Of 94 pT ≥ 3a prostatic lesions, 45.7% (*n* = 43) were detected (miT ≥ 3a) by ^68^Ga-PSMA-11 PET. 80.4% (*n* = 74) of pT2 prostatic lesions were correctly classified as miT2. Cross tables are presented in Additional file 1: Tables S1 and S2.

In the univariate analysis (Additional file 1: Table S3), a significant association was detected between a positive surgical margin and the following parameters: high miT status (miT ≥ 3a, OR: 3.38, *p* = 0.004), miN1 status (OR: 7.526, *p* < 0.001), SUV_max_ (OR: 1.026, *p* = 0.039) and TL (OR: 1.007, *p* = 0.021). In the multivariate analysis (Table 4), miN1 (OR: 5.428, *p* = 0.004) was significantly associated with a positive surgical margin. Moreover, a significant association was present between miT ≥ 3a and GS ≥ 8 (OR: 2.696, *p* = 0.003) (Table 5).

**Table 4:** Multivariate analysis for the association of ^68^Ga-PSMA-11 PET findings with surgical margin status

	No. of patients	Odds ratio	95% CI	*p* value*
miTNM classification, no., *n* = 186				
miT status				
2	125	Reference		
≥ 3a	61	2.065	0.802–5.315	0.133
miN status				
No LN metastasis	168	Reference		
With LN metastasis	18	5.428	1.708–17.249	**0.004**
SUV_max_ of prostatic lesions	183	1.015	0.988–1.044	0.282
TL of prostatic lesions	183	1.004	0.998–1.011	0.166

*CI* confidence interval, *LN* lymph node, *PET* positron emission tomography, *PSMA* prostate-specific membrane antigen, *SUV* standardized uptake value, *TL* total lesion, *TV* tumor volume

*Significant associations are given in bold

**Table 5:** Univariate analysis for the association of ^68^Ga-PSMA-11 PET findings with Gleason Score

	No. of patients	Odds ratio	95% CI	*p* value*
miTNM classification, no., *n* = 186				
miT status				
2	125	Reference		
≥ 3a	61	2.696	1.39–5.23	**0.003**
miN status				
No LN metastasis	168	Reference		
With LN metastasis	18	2.187	0.812–5.887	0.122
SUV_mean_ of prostatic lesions	183	1.020	0.986–1.056	0.248
SUV_max_ of prostatic lesions	183	1.017	0.995–1.040	0.138
TV of prostatic lesions	183	0.981	0.941–1.022	0.353
TL of prostatic lesions	183	1.006	1–1.012	0.056

*CI* confidence interval, *LN* lymph node, *PET* positron emission tomography, *PSMA* prostate-specific membrane antigen, *SUV* standardized uptake value, *TL* total lesion, *TV* tumor volume

*Significant associations are given in bold

### Predictors of BCR-free survival

**Fig. 1:**
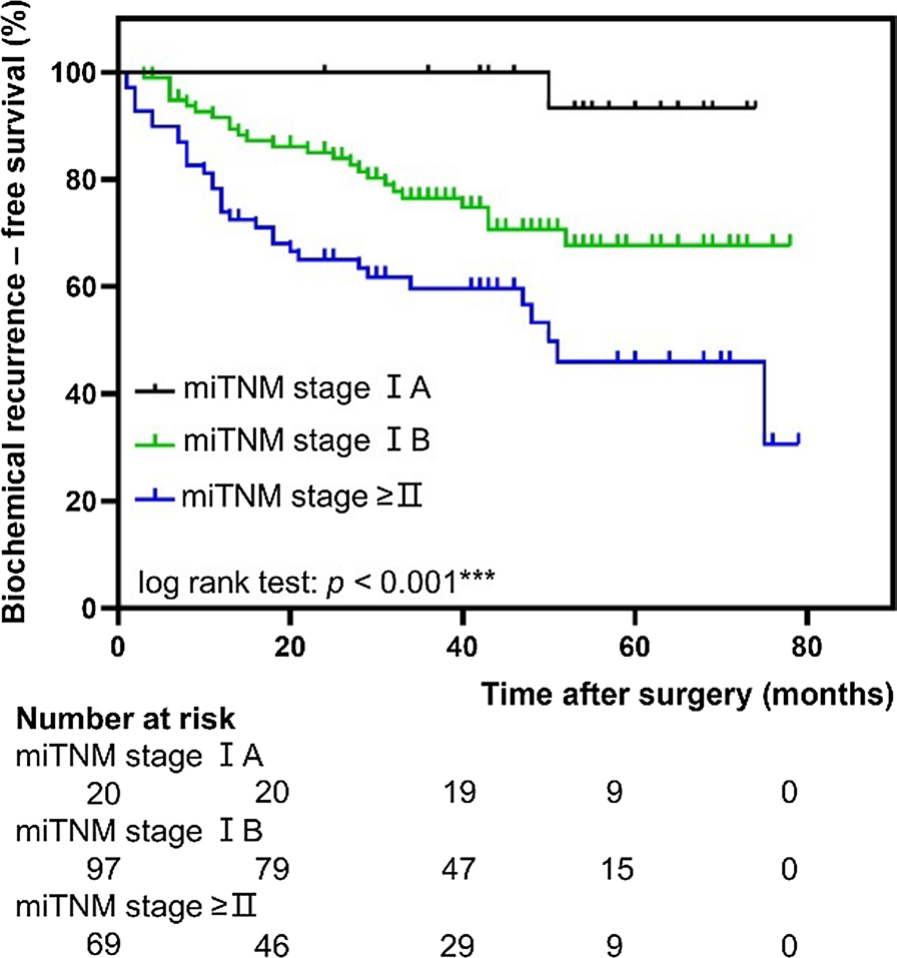
Biochemical recurrence-free survival according to miTNM stage. Pairwise comparison: miTNM stage IA versus miTNM stage IB, *p* = 0.022; miTNM stage IA versus miTNM stage ≥ II, *p* = 0.001; miTNM stage IB versus miTNM stage ≥ II, *p* = 0.005

**Fig. 2:**
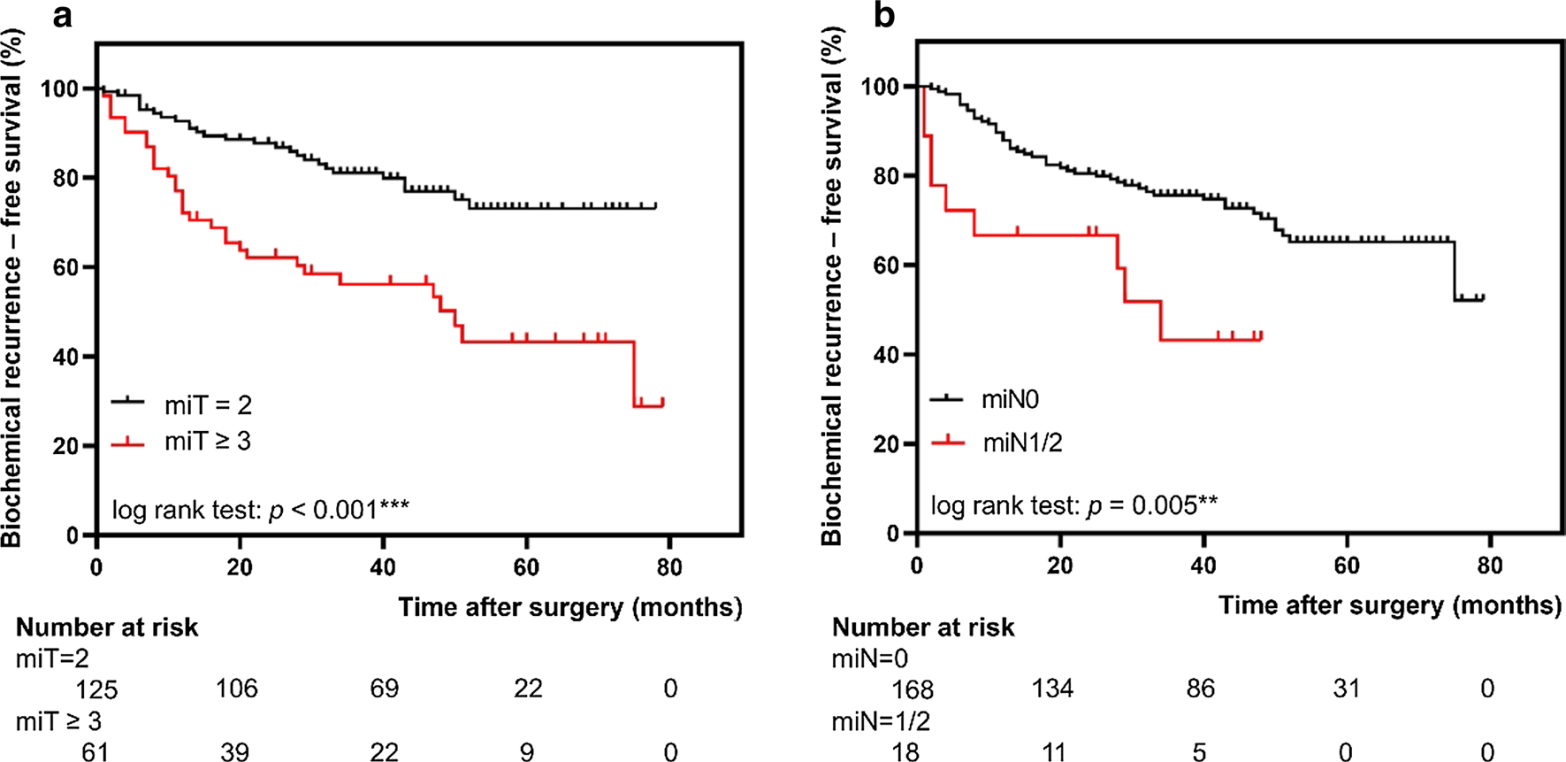
Longer biochemical recurrence-free survival was associated with **a** miT = 2 and **b** miN = 0

**Fig. 3:**
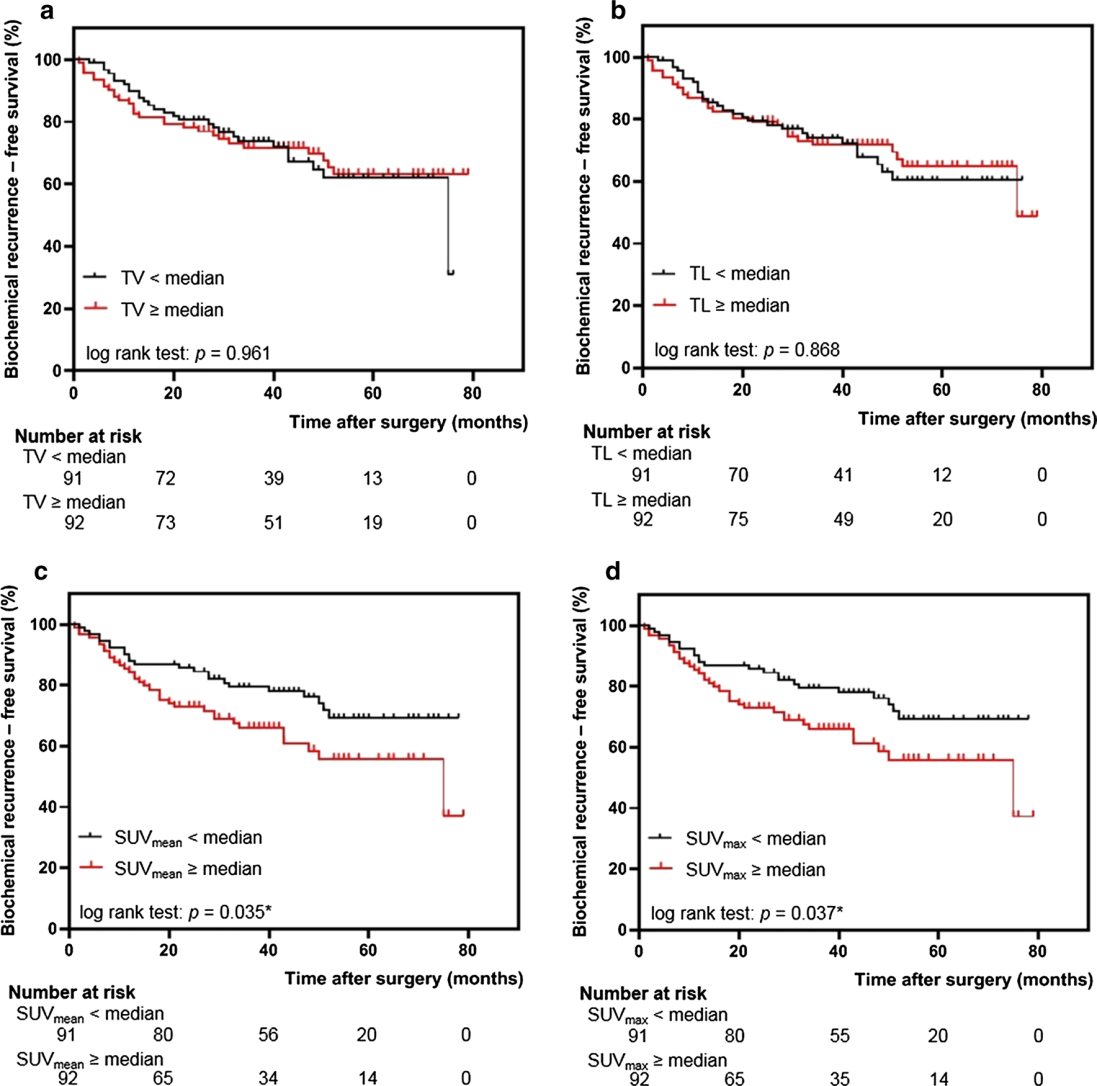
Kaplan–Meier curves comparing biochemical recurrence-free survival of selected patients stratified by **a** tumor volume, **b** total lesion, **c** SUV_mean_ and **d** SUV_max_. Longer biochemical recurrence-free survival was associated with lower SUV_mean_ and SUV_max_

Kaplan–Meier curves for BCR-free survival with different clinicopathological and miTNM-derived parameters are shown in Figures 1, 2, 3 and Additional file 1: Fig. S2.

The miTNM derived parameters miT2 versus miT3 disease or higher (log-rank, *p* < 0.001, Fig. 2a), miN0 versus miN1/2 (log-rank, *p* = 0.005, Fig. 2b) and miTNM stage group IA compared with miTNM stage group IB (log-rank, *p* = 0.022, Fig. 1) were associated with significantly different BCR-free survival rate. Lower SUV_mean_ and SUV_max_ as quantitative parameters from ^68^Ga-PSMA-11 PET were also associated with longer BCR-free in patients (Fig. 3c, d, log-rank, *p* = 0.035, *p* = 0.037, respectively).

The following pathological features were associated with longer BCR-free survival: pT2 versus ≥ pT3a (log-rank, *p* < 0.001, Additional file 1: Fig. S2A). pN0 versus pN1 (log-rank, *p* < 0.001, Additional file 1: Fig. S2B), lower Gleason Grades (GS < 8) (log-rank, *p* < 0.001, Additional file 1: Fig. S2C) and negative surgical margins (log-rank, *p* < 0.001, Additional file 1: Fig. S2D).

Results from a univariate Cox regression analysis investigating preoperative and postoperative risk factors for BCR is presented in Table 6. We found that following factors were significantly associated with BCR-free survival in prostate cancer patients: clinical data including age (HR: 1.056, 95% CI 1.018–1.096, *p* = 0.004) and initial PSA (iPSA) (HR: 1.021, 95% CI 1.007–1.035, *p* = 0.003); pathological data including Gleason score (GS ≥ 8 vs. GS < 8, HR: 5.097, 95% CI 3.013–8.625, *p* < 0.001), pT stage (pT ≥ 3 vs. pT < 3, HR: 2.935, 95% CI 1.665–5.173, *p* < 0.001), pN stage (pN1 vs. pN0, HR: 3.378, 95% CI 1.901–6, *p* < 0.001) and surgical margin (positive vs. negative, HR: 3.421, 95% CI 1.890–6.193, *p* < 0.001). Imaging parameters include miT stage (miT ≥ 3a vs. miT < 3, HR: 2.811, 95% CI 1.673–4.722, *p* < 0.001), miN stage (miN1 vs. miN0, HR: 2.691, 95% CI 1.311–5.527, *p* = 0.007), SUV_mean_ of prostatic lesions (HR: 1.019, 95% CI 1.002–1.036, *p* = 0.028), SUV_max_ of prostatic lesions (HR: 1.015, 95% CI 1.004–1.026, *p* = 0.008) and TV of prostatic lesions (HR: 0.948, 95% CI 0.909–0.988, *p* = 0.011).

**Table 6:** Univariable analysis for the association of baseline factors with BCR-free survival

	No. of patients	Hazard ratio	95% CI	*p* value*
Clinical data				
Age	186	1.056	1.018–1.096	**0.004**
iPSA	184	1.021	1.007–1.035	**0.003**
Pathological data				
Gleason score in surgical specimen, no., *n* = 186				
6–7	133	Reference		
8–10	53	5.097	3.013–8.625	**< 0.001**
Pathological stage, no., *n* = 186				
pT status				
2	92	Reference		
≥ 3	94	2.935	1.665–5.173	**< 0.001**
pN status				
0	154	Reference		
1	32	3.378	1.901–6.000	**< 0.001**
Surgical margin, no., *n* = 180				
Negative	152	Reference		
Positive	28	3.421	1.890–6.193	**< 0.001**
Imaging parameters				
miTNM classification, no., n = 186				
miT status				
2	125	Reference		
≥ 3a	61	2.811	1.673–4.722	**< 0.001**
miN status				
No LN metastasis	168	Reference		
With LN metastasis	18	2.691	1.311–5.527	**0.007**
SUV_mean_ of prostatic lesions	183	1.019	1.002–1.036	**0.028**
SUV_mean_ of prostatic lesions, no. *n* = 183				
< median	91	Reference		
≥ median	92	1.752	1.030–2.981	**0.039**
SUV_max_ of prostatic lesions	183	1.015	1.004–1.026	**0.008**
SUV_max_ of prostatic lesions, no. *n* = 183				
< median	91	Reference		
≥ median	92	1.744	1.025–2.968	**0.040**
TV of prostatic lesions	183	0.948	0.909–0.988	**0.011**
TV of prostatic lesions, no., *n* = 183				
< median	91	Reference		
≥ median	92	0.987	0.587–1.661	0.962
TL of prostatic lesions	183	1.003	1.000–1.006	0.072
TL of prostatic lesions, no., *n* = 183				
< median	91	Reference		
≥ median	92	0.957	0.568–1.612	0.869

*BCR* biochemical recurrence, *CI* confidence interval, *iPSA* initial PSA, *IQR* interquartile range, *LN* lymph node, *PET* positron emission tomography, *PSA* prostate-specific antigen, *PSMA* prostate-specific membrane antigen, *SUV* standardized uptake value, *TL* total lesion, *TV* tumor volume

*Significant associations are given in bold

In the multivariate Cox regression analysis (Table 7), the following factors were independent predictors for BCR-free survival: serum PSA level (HR: 1.024, 95% CI 1.005–1.043, *p* = 0.014), advanced pathological Gleason Score (GS ≥ 8 vs. GS < 8, HR: 3.253, 95% CI 1.779–5.950; *p* < 0.001) and miT stage (miT ≥ 3a vs. miT < 3, HR: 1.941, 95% CI 1.047–3.599, *p* = 0.035).

**Table 7:** Multivariable analysis for the association of baseline factors with BCR-free survival

	No. of patients	Hazard ratio	95% CI	*p* value*
Clinical data				
Age, no., *n* = 186				
Continuous		1.030	0.991–1.071	0.133
iPSA, no., *n* = 184				
Continuous		1.024	1.005–1.043	**0.014**
Pathological data				
Gleason score in surgical specimen, no., *n* = 186				
6–7	133	Reference		
8–10	53	3.253	1.779–5.950	** < 0.001**
pT status, no., *n* = 186				
2	92	Reference		
3	94	1.471	0.773–2.797	0.239
pN status, no., *n* = 186				
No LN metastasis	154	Reference		
With LN metastasis	32	1.027	0.418–2.525	0.954
Surgical margin, no., *n* = 180				
Negative	152	Reference		
Positive	28	1.539	0.716–3.305	0.269
Imaging parameters				
miT status from PSMA PET, no., *n* = 186				
2	125	Reference		
≥ 3a	61	1.941	1.047–3.599	**0.035**
miN status from PSMA PET, no., *n* = 186				
No LN metastasis	168	Reference		
With LN metastasis	18	1.233	0.389–3.908	0.722
SUV_mean_, no., *n* = 183				
Continuous		0.743	0.491–1.123	0.159
SUV_max_, no., *n* = 183				
Continuous		1.202	0.943–1.532	0.137
TV				
Continuous		0.934	0.883–0.988	**0.017**

*BCR* biochemical recurrence, *CI* confidence interval, *iPSA* initial PSA, *LN* lymph node, *PET* positron emission tomography, *PSA* prostate-specific antigen, *PSMA* prostate-specific membrane antigen, *SUV* standardized uptake value, *TV* tumor volume

*Significant associations are given in bold

## Discussion

Our retrospective analysis demonstrates the potential predictive value of ^68^Ga-PSMA-11 PET findings for BCR-free survival of primary prostate cancer after RP. Information from the recently introduced miTNM classification is associated with outcome. In addition, several correlations between quantitative and qualitative measures from ^68^Ga-PSMA-11 PET and pathological parameters have been observed. Prognostic tools of BCR are required and essential for improving treatment management of prostate cancer patients and reducing prostate cancer-associated mortality of patients developing BCR after primary treatment [32]. With the successful application of PSMA-ligand PET for primary staging in prostate cancer patients, clinical studies are necessary to investigate its predictive value. PSMA-ligand PET is increasingly used for selection, monitoring and individualization of prostate cancer treatments.

The present analysis is the first to investigate the association of miTNM classification from preoperative ^68^Ga-PSMA-11 PET imaging and postoperative histopathological findings and the potential of miTNM reporting to serve as predictors for BCR after RP. Consequently, we performed a prognostic validation of the miTNM system as a framework for PSMA-ligand PET reporting in a relatively large patient cohort. Preoperative ^68^Ga-PSMA-11 PET and miTNM classification could help to stratify risk for BCR after RP and could potentially further influence the clinical patient management.

Previous studies have extensively assessed the predictors of BCR. Clinicopathological characteristics, including pathological aggressive GS [5–9], positive nerve invasion [8], pathological T stage [7] and preoperative PSA [3–6], were proven to have a strong association with BCR. Our present results are in accordance with literature data. The data from our analysis outline serum PSA level (HR: 1.024, 95% CI 1.005–1.043, *p* = 0.014) and advanced pathological Gleason score (GS ≥ 8 vs. GS < 8, HR: 3.253, 95% CI 1.779–5.950; *p* < 0.001) as important histopathological predictors of BCR.

In addition, and novel compared to the current literature our data indicate that also parameters from ^68^Ga-PSMA-11 PET might serve as non-invasive biomarkers. We identified a miT stage ≥ 3a in ^68^Ga-PSMA-11 PET as surrogate for higher GS (OR: 2.696, 95% CI 1.39–5.23, *p* = 0.003) and worse BCR-free survival (HR: 1.941, 95% CI 1.047–3.599, *p* = 0.035). Notably, pelvic lymph node metastases in ^68^Ga-PSMA-11 PET were not detected as an independent predictor for BCR in this study (HR: 1.233, 95% CI 0.389–3.908, *p* = 0.722). However, the BCR-free survival differed significantly between miN0 and miN1 group (log-rank, *p* = 0.005). Interestingly also Raheem et al. have failed to detect lymph nodes in histopathology as an independent predictor to BCR after RP in a study including 359 patients [4]. Of note, the sample size of miN1 group (*n* = 18) in our cohort was relatively small. Thus, the interpretation of results should be with caution, and further studies including more miN1 patients are needed to clarify the predictive value of miN classification for BCR after RP.

Besides, our results indicate a negative association of TV with BCR-free survival (HR: 0.934, 95% CI 0.883–0.988, *p* = 0.017). Contrarily, Choi et al. have reported a significantly higher BCR-free survival rate in pT2 prostate cancer patients with percent tumor volume ≤ 7.5%, which was assessed using histological samples (*p* < 0.001) [33]. This is partly related to the methods of obtaining tumor volume and studies are necessary to assess the standard of TV calculation from ^68^Ga-PSMA-11 PET and pathological samples.

With this work we also introduced a stage grading system based on the recent proposed molecular staging system (miTNM staging system, version 1.0) combined with quantitative parameters. It is intended to mirror the AJCC staging system based on clinicopathological parameters which has proven to be a fundamental tool that also informs treatment decisions [34]. Bhindi et al. have confirmed the ability of the 8^th^ edition to predict oncologic outcomes [35]. However, the AJCC staging system utilized clinical or pathological TNM stage and no parameters from imaging. With the increasing use of PSMA-ligand PET in clinical routine, a logical next step is to use information from non-invasive imaging prior to definite treatment for risk stratification.

We have shown that the miT stage is an independent predictor of BCR, and we observed a widely varying prognosis in the miT2 stage patients. Similarly, a recent study has revealed that high intraprostatic ^68^Ga-PSMA-11 uptake (SUV_max_ > 8) predicts short progression-free survival rate among patients with GS 3 + 4 on biopsy [36]. The significant difference in BCR-free survival rate has been confirmed in IA and IB stage groups. Our findings propose that a SUV_max_ cut-off extracted from literature could further stratify the group of miT2 primary disease into patients with more aggressive disease and worse prognosis. Further studies are necessary for prognostic validation of other stage groups.

The present study has several limitations. It is a retrospective analysis and includes only patients from a single center, which can introduce potential bias. Despite inclusions of a large number of patients, the sample size of patients in the miM1 group was too small to conduct meaningful analysis. This is mainly related to the fact that most patients with extrapelvic metastases do not undergo primary curative RP but either get systemic treatment with or without local treatment. This explains the low number of patients in miTNM stage group III and IV. In summary, further prospective investigations with large patient numbers are necessary to fully investigate the potential of the miTNM staging and our proposed grading system to predict patient outcome after curative intent RP.

## Conclusion

Our retrospective analysis indicates that the miTNM framework developed to standardize PSMA-ligand PET reported is independently associated with BCR-free survival of primary prostate cancer after RP. We demonstrated significant associations between ^68^Ga-PSMA-11 PET findings and histopathological parameters. In summary, our results outline that the miTNM classification and the presented further development of a miTNM-based stage group system can serve as non-invasive imaging biomarkers of risk stratification for primary prostate cancer patients. However, further and prospective studies including patients with different treatments and stages are needed to fully assess the predictive value of PSMA-ligand PET imaging in the setting of newly diagnosed prostate cancer.

## Supplementary Information

**Additional file 1**. Supplementary files. **Supplementary table 1.** Distribution of pT and miT. **Supplementary table 2.** Distribution of pN and miN. **Supplementary table 3.** Univariate analysis for the association of ^68^Ga-PSMA-11 PET findings with surgical margin status. **Supplementary Fig. 1.** Flowchart of inclusion and exclusion steps. **Supplementary Fig. 2.** Longer biochemical recurrence-free survival was associated with (A) pT = 2, (B) pN=0, (C) Gleason Score < 8 and (D) negative surgical margin.

## Data Availability

The datasets generated during and/or analyzed during the current study are available from the corresponding author on a reasonable request.

## Abbreviations

^68^Ga: Gallium-68
AJCC: American Joint Committee on Cancer
BCR: Biochemical recurrence
BSI: Bone scan index
^11^C: Carbon-11
CI: Confidence interval
CT: Computed tomography
DNA: Deoxyribonucleic acid
ESCC: Esophageal squamous cell carcinoma
FDG: Fluorodeoxyglucose
GS: Gleason score
HR: Hazard ratio
iPSA: Initial PSA
IQR: Interquartile range
mCRPC: Metastatic castration-resistant prostate cancer
miTNM: Imaging TNM classification
MRI: Magnetic resonance imaging
OR: Odds ratio
PET: Positron emission tomography
PSA: Prostate-specific antigen
PSMA: Prostate-specific membrane antigen
RP: Radical prostatectomy
SD: Standard deviation
SUV: Standardized uptake value
TL: Total lesion
TV: Tumor volume
VOI: Volume of interest
