# Supplementary material for: PSMA-ligand uptake can serve as a novel biomarker in primary prostate cancer to predict outcome after radical prostatectomy
Source: EJNMMI Res. 2021 Aug 21;11:76. doi: 10.1186/s13550-021-00818-2 (PMC8380207; doi:10.1186/s13550-021-00818-2)

Supplementary table 1. Distribution of pT and miT.

|  | pT2 | pT ≥ 3a | Total |
| --- | --- | --- | --- |
| miT2 | 74 | 51 | 125 |
| miT ≥ 3a | 18 | 43 | 61 |
| Total | 92 | 94 | 186 |

Supplementary table 2. Distribution of pN and miN.

|  | pN0 | pN1 | Total |
| --- | --- | --- | --- |
| miN0 | 149 | 19 | 168 |
| miN1/2 | 5 | 13 | 18 |
| Total | 154 | 32 | 186 |

Supplementary table 3. Univariate analysis for the association of ^68^Ga-PSMA-11 PET findings with surgical margin status.

|  | No. of  patients | Odds ratio | 95% CI | *p* value* |
| --- | --- | --- | --- | --- |
| miTNM classification, no., n = 186 | | | | |
| miT status | | | | |
| 2 | 125 | Reference |  |  |
| ≥ 3a | 61 | 3.380 | 1.477-7.732 | **0.004** |
| miN status | | | | |
| No LN metastasis | 168 | Reference |  |  |
| With LN metastasis | 18 | 7.526 | 2.659-21.305 | **<0.001** |
| SUV_mean_ of prostatic lesions | 183 | 1.035 | 0.996-1.076 | 0.076 |
| SUV_max_ of prostatic lesions | 183 | 1.026 | 1.001-1.052 | **0.039** |
| TV of prostatic lesions | 183 | 0.992 | 0.941-1.046 | 0.775 |
| TL of prostatic lesions | 183 | 1.007 | 1.001-1.014 | **0.021** |

*Significant associations are given in bold.

CI = confidence interval; LN = lymph node; PET = positron emission tomography; PSMA = prostate-specific membrane antigen; SUV = standardized uptake value; TL = total lesion; TV = tumor volume.

Supplementary Fig.1 Flowchart of inclusion and exclusion steps.


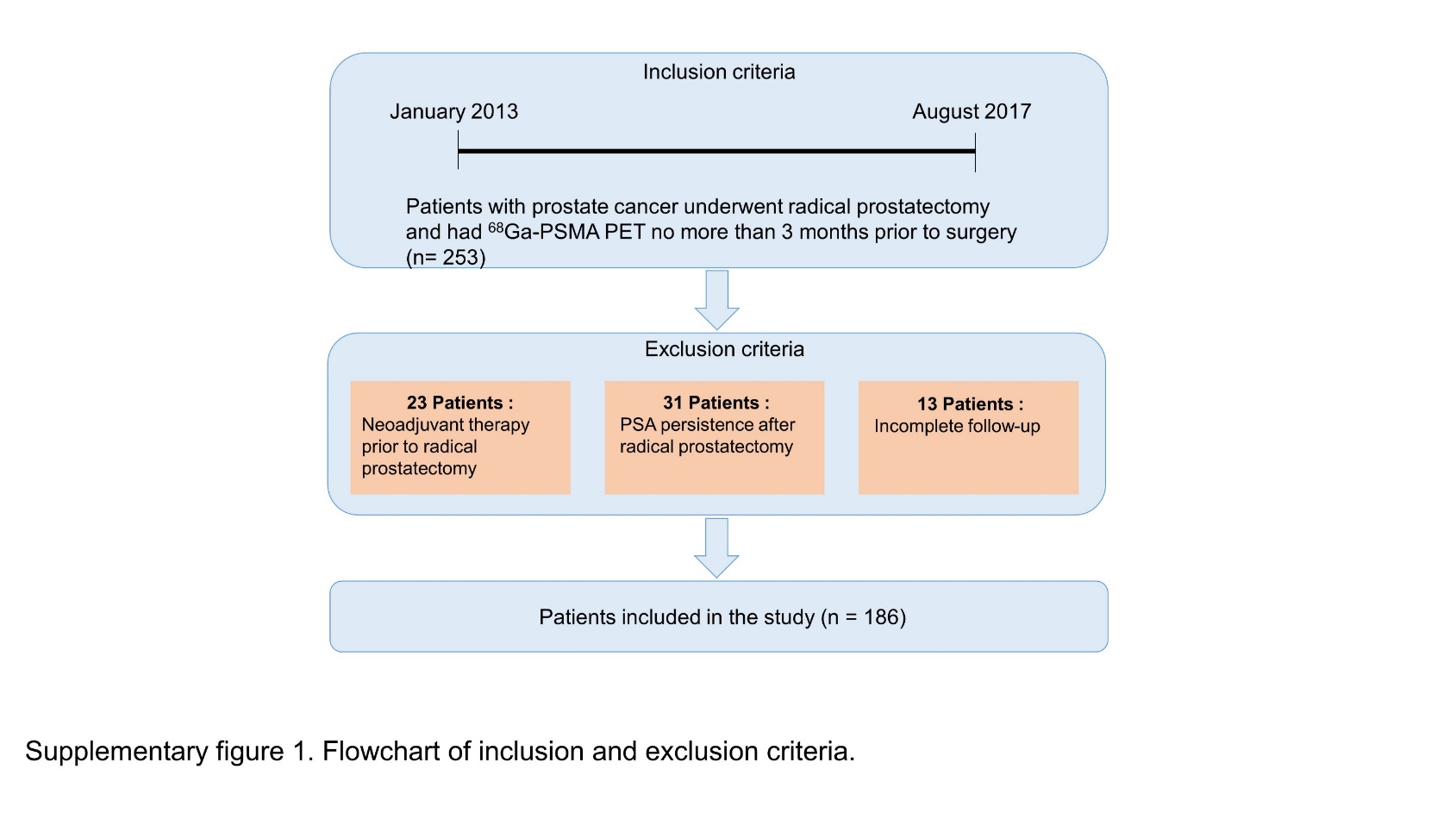


Supplementary Fig.2 Longer biochemical recurrence-free survival was associated with (A) pT=2, (B) pN=0, (C) Gleason Score < 8 and (D) negative surgical margin.


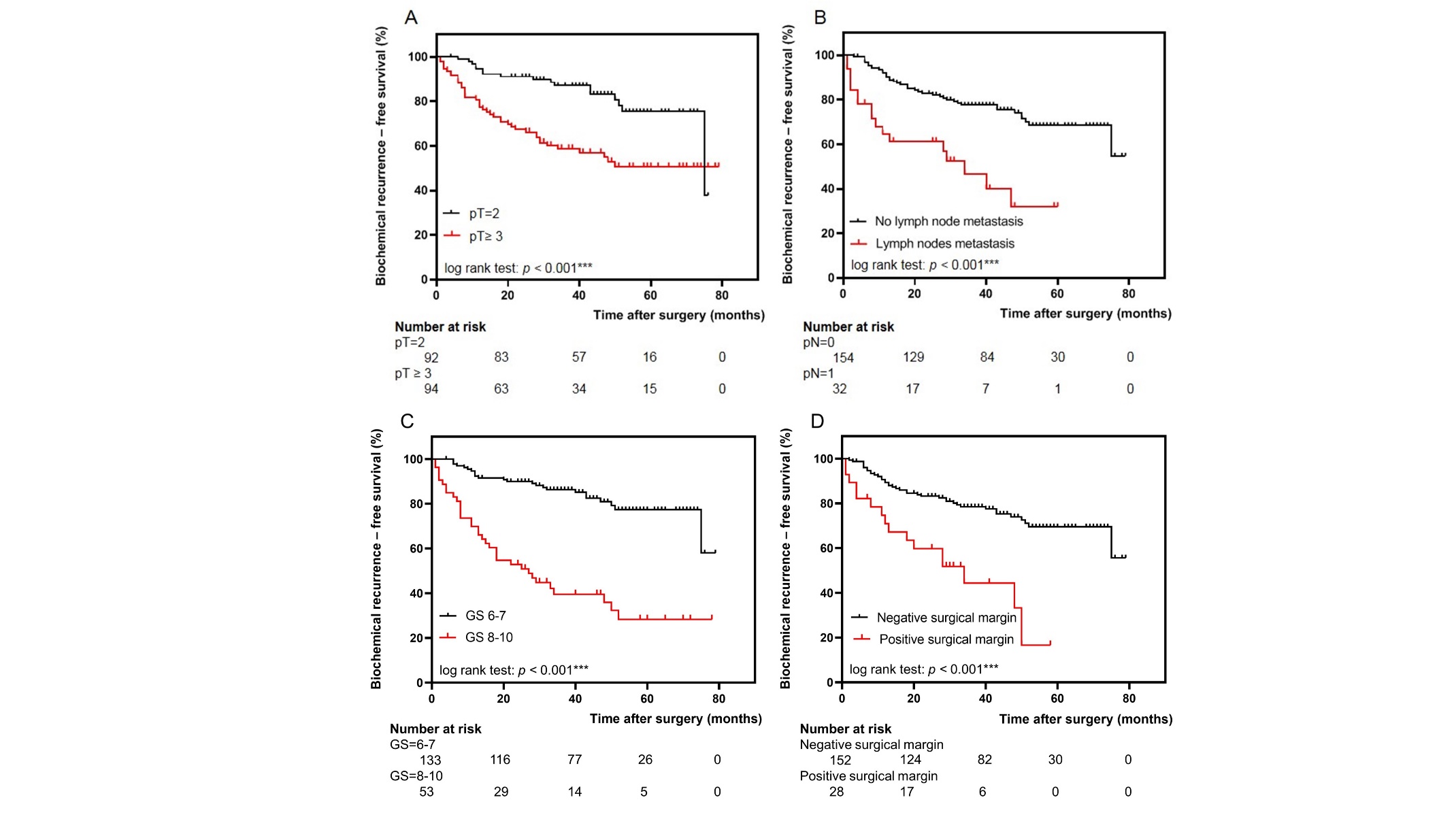

Supplement: Supplementary file 1 — Additional file 1. Supplementary files. Supplementary table 1. Distribution of pT and miT. Supplementary table 2. Distribution of pN and miN. Supplementary table 3. Univariate analysis for the association of 68Ga-PSMA-11 PET findings with surgical margin status. Supplementary Fig. 1. Flowchart of inclusion and exclusion steps. Supplementary Fig. 2. Longer biochemical recurrence-free survival was associated with (A) pT = 2, (B) pN=0, (C) Gleason Score < 8 and (D) negative surgical margin. [file 13550_2021_818_MOESM1_ESM.docx]
